# Mating competition among females: testing the distinction between natural and sexual selection in an insect

**DOI:** 10.1098/rsos.240191

**Published:** 2024-04-03

**Authors:** Varpu Pärssinen, Leigh W. Simmons, Charlotta Kvarnemo

**Affiliations:** ^1^ Department of Biological and Environmental Sciences, University of Gothenburg, Gothenburg 40530, Sweden; ^2^ Centre for Evolutionary Biology, School of Biological Sciences (M092), The University of Western Australia, Crawley 6009, Australia

**Keywords:** multiple mating, natural selection, spermatophore, polyandry, sexual selection

## Abstract

In species where females compete for mates, the male often provides the female with resources in addition to gametes. A recently suggested definition of sexual selection proposed that if females only benefit from additional resources that come with each mating and not additional gametes, female intrasexual competition for mating opportunities would result in natural selection rather than sexual selection. The nuptial gift-giving bushcricket *Kawanaphila nartee* has dynamic sex roles and has been a textbook example of sexual selection acting on females via mating competition. We investigated whether females of this species gain fitness benefits from nuptial gifts, additional ejaculates or both by controlling the number of matings and whether the female was allowed to consume the nutritious gift (spermatophylax) at mating. We found that egg production per day of life increased with the number of additional matings, both with and without spermatophylax consumption, but consuming the spermatophylax had an additional positive effect on the number of eggs. These effects were particularly strong in females with shorter lifespans. We discuss how the recently suggested definition of sexual selection applies to nuptial-feeding insects and conclude that both natural and sexual selections influence mating competition in *K. nartee* females.

## 1. Introduction

The extent to which sexual selection acts on females has been contentious ever since the concept was first introduced [1–3]. While the study of sexual selection has historically focused on male–male mating competition, we now know that females can also compete with other females over mating opportunities, especially when the males provide the female with additional direct benefits such as parental care or other resources [3–7]. However, as we have gained more knowledge about different forms of mate choice and mating competition, the boundary between sexual selection and natural selection has become harder to define [8,9]. To help clarify the issues, Shuker & Kvarnemo [10] recently proposed a new definition, which limits sexual selection to fitness gains associated with the competition for access to gametes for fertilization. Under this definition, any intrasexual competition that occurs only to secure resources provided by the opposite sex will fall under natural selection. Consequently, the proposed definition questions whether many instances of female mating competition should still be considered sexual selection.

Female animals are often assumed to be less mate-limited than males by default, as males can produce more gametes than females and thus maximize their fitness by fertilizing eggs of multiple females [11–14]. However, this assumption is not always true in nature, where the potential reproductive rate of males can be restricted by environmental variation or high paternal investment [15–18]. Indeed, in a majority of mating systems where sexual selection has been thought to act on females, the males provide the females with resources other than gametes, such as nesting sites, parental care or nuptial gifts [3,4]. In some of these mating systems, the low availability of males may leave some females unable to access high-quality males or even fail to mate at all [19], and so they are best interpreted as being subject to sexual selection [10]. However, in many other mating systems, virtually all females mate at least once, but they continue to compete over chances to mate with multiple males. In such cases of polyandry, competition among females may occur in order to gain sperm from multiple males or to acquire more resources from those males.

Females can gain fitness benefits from multiple mating in at least two ways. First, access to more sperm, especially from multiple males, can increase the number or quality of their offspring [20–22]. For example, even females that store sperm may need to replenish depleted sperm stores to maximize their reproductive output [23]. The average fitness of a female’s offspring may also be improved by increased genetic diversity from being fathered by multiple males [24–27]. In these cases, competition among females for gametes is best interpreted as sexual selection, regardless of whether the females gain direct benefits (replenished sperm stores) or indirect benefits (genetic diversity of offspring) from each mating. Second, each mating might give the female additional direct benefits, such as nutrition that can be allocated to producing more offspring. In insect species where the male provides the female with an edible nuptial gift during mating, the evidence from several taxa shows that the compounds contained in the nuptial gift can be used in the production of offspring [28–31]. However, if multiple mating only increased female fitness through the additional resources and not additional sperm, female competition for access to mates should be considered resource competition and, thus, better interpreted as natural selection. The main argument for this view is that gaining access to nutrients by mating with multiple males is simply an alternative way of foraging [32]. Disentangling the benefits derived from additional gametes and nutritional resources would bring us closer to understanding why female mating competition typically evolves, and the extent to which females are subject to sexual selection versus natural selection for increased mating frequency [10].

The benefits of polyandry have been evaluated in many nuptial-feeding species, but few studies have attempted to partition the effects of additional sperm and additional nutrition on female fecundity. The reviews by Arnqvist & Nilsson [33] and South & Lewis [20] conclude that multiple mating generally boosts egg production, whether nuptial feeding is present in the species or not. Consuming multiple nuptial gifts without additional mating has also been shown to increase female fecundity in several species [34–36], especially in resource-limited conditions. On the other hand, studies manipulating food levels between mating treatments have generally yielded mixed results, showing that edible gifts may not always function as a replacement food source, depending on species [33,37,38]. The few studies that use experimentally controlled matings with or without a nuptial gift have not evaluated the importance of nuptial feeding beyond the first mating [37,39], or did not find fitness benefits from nuptial feeding even on the first mating [39,40]. Thus, it remains unclear if the benefits from multiple mating originate from gaining access to additional gametes or additional nuptial food gifts, which is the question at the heart of this study.

The Australian bushcricket *Kawanaphila nartee* (Orthoptera: Tettigoniidae) is a model organism in the study of female–female mating competition, which is rarely described in other bushcrickets and is often cited as an example of sexual selection acting on females [18,41,42]. Upon mating, the male provides the female with a protein-rich nuptial gift in the form of a spermatophylax, which is attached to the sperm-containing ampulla of the spermatophore and consumed by the female while sperm are transferred to her reproductive tract [43]. Consumption of the spermatophylax provides nutrient resources that are used in egg production, and the spermatophore and its associated spermatophylax represent 20% of a male’s body mass [43,44]. Males need to feed on pollen for several days to produce a spermatophore, so the low availability of pollen in early spring limits the number of males available for mating. As a result, females of *K. nartee* compete for access to mates in the first half of the breeding season [45]. Females also seem to gain more fitness benefits from spermatophylax consumption during this resource-limited period [45]. Although a recent study suggested that Bateman gradients for female *K. nartee* were close to zero, these gradients were based on point estimates of female fecundity, and it remains unclear how multiple mating affects female lifetime fitness [46].

In this study, we critically evaluate our previous characterization of female mating competition in *K. nartee* as sexual selection and whether it should fall under resource competition (and thus, natural selection) instead. To achieve this, we experimentally examine the potential fitness benefits of multiple mating in female *K. nartee*. We predict that female fitness (in terms of lifetime egg production, average egg weight and egg viability) will increase with additional matings. If female fitness increases from multiple mating when females receive gametes but are prevented from consuming the nuptial gift, sexual selection can be inferred as acting on female mating frequency for the acquisition of gametes. By contrast, if female fitness is elevated only when they are also allowed to consume the nuptial gift component of the spermatophore, natural selection can be inferred as acting on female mating frequency for the acquisition of nutrient resources.

## 2. Material and methods

### 2.1. Study system

*Kawanaphila nartee* is a flightless, nocturnal Australian bushcricket that feeds exclusively on the pollen and nectar of spring flowering plants [18]. The nymphs hatch in mid-winter (July) and reach adulthood in the early spring (late August). Breeding occurs throughout spring (September–November), during which time the insects feed on pollen and nectar from a variety of flowering plants. At the start of the breeding season, *K. nartee* feed mostly on the flowers of kangaroo paws (*Anigozanthos manglesii*) and switch to the more pollen-rich flowers of grasstrees (*Xanthorrhoea preissei*) once they start blooming in mid to late October. Adults perish in November dependent on the onset of the first hot dry days of summer. Eggs laid during the spring remain dormant in the soil until the rains return the following winter [18].

The sex roles of *K. nartee* vary dynamically, following the seasonal changes in resource abundance in their environment [18,45,47]. In the early season, when feeding on kangaroo paws, females compete over mating opportunities, sometimes physically grappling with each other over the chance to mount a calling male [48], while males frequently reject mating attempts from females [45]. By contrast, when the grasstrees flower, pollen and nectar become available in abundance, the number of sexually active males is higher compared with females that are receptive to mating, and males compete acoustically for choosy females [45]. Evidence of strong selection acting on females has come from studies that show sexual dimorphism in their hearing morphology. Females have larger auditory spiracles, and this trait appears to be under positive directional selection because females with larger ear openings are able to locate calling males faster [48–50], which is particularly important during the early half of the breeding season when the availability of males is limited [49]. By the end of the breeding season, almost all females have mated with several males [49].

Females initiate mating by climbing onto the male’s back and locking the male into a genital hold (*precopula*). If the male does not reject the female and the mating proceeds, he will transfer a spermatophore, consisting of a sperm-containing ampulla surrounded by a gelatinous spermatophylax [18,51]. The pair then separates and the female bends forward to eat the spermatophore that is attached to her genital opening. Sperm transfer from the ampulla occurs during the time that the female is feeding on the spermatophylax [52]. After having eaten the spermatophylax, the female ingests the emptied ampulla. Consuming the spermatophylax increases gametic mass, number of eggs and the average weight of eggs produced by the female up to 72 h after a single mating [43]. Furthermore, radioisotope labelling has shown that nutrients contained in the spermatophylax are allocated into developing eggs [44]. The effects of spermatophylax consumption beyond the first mating, as well as potential benefits gained from multiple ejaculates, have not yet been evaluated in this species. Compounds in the ejaculate also affect the female refractory period after a mating, during which females are unreceptive to remating [52]. However, while the refractory period can be as long as 20 days after the first mating, it is significantly shorter, as short as 4 days, if the female has limited food resources and is prevented from eating the spermatophylax [52]. This suggests that female choice to remate early is at least partly motivated by resource acquisition.

### 2.2. Collection and housing

**Figure 1.**
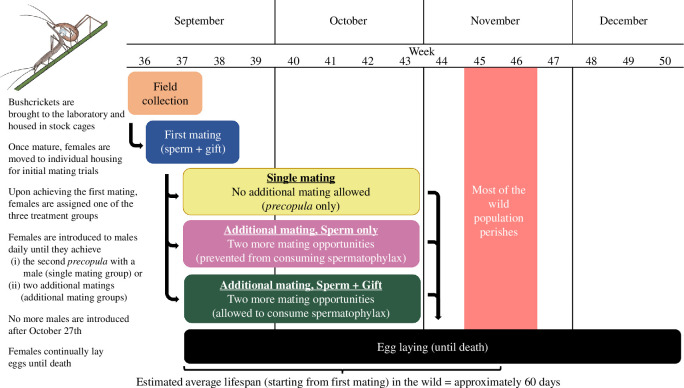
Timeline and experimental design of the study.

*Kawanaphila nartee* were collected from King’s Park, Perth, Western Australia, between 5 and 13 September 2022 (figure 1). At the time of collection, the majority of females still had not completed their final moult and thus were not yet sexually mature. A separate evaluation found that only 5% of adult females were mated in the first week after adult individuals are first found in the population (Pärssinen V, Kvarnemo C & Simmons LW 2023,unpublished data), so the few adult females that were collected were presumed to be unmated. Both adult and juvenile bushcrickets were separated by sex (females are clearly recognizable from their ovipositor even as nymphs) and held in fly screen cages in a constant temperature room with a 12 L : 12 D photoperiod and a temperature regime of 18°C : 12°C. The cages were supplied with fronds from grasstrees for perching and a box of wet cotton wool for access to water, and the cages were misted with water at least once each day. The juvenile cages were checked daily for emerging adults, which were transferred to adult-holding cages. Males were provided with pollen ad libitum that was replenished regularly to ensure that they would be able to produce a spermatophore, whereas adult females received limited rations of pollen to mimic early spring conditions in the wild. Pollen was obtained from a health food store and ground to a fine powder. It was provided to the bushcrickets adhered to a dried grasstree flower stem that had been dipped first in a saturated solution of glucose and then rolled in ground pollen.

### 2.3. Experimental procedure

#### 2.3.1. Individual housing

Our experimental set-up had the capacity to house 140 females. Recently emerged adult females were taken from the stock cages and placed individually in 400 ml plastic vials with net-covered openings for ventilation. Each container had a single cardboard cup, made of a disassembled egg carton, that was filled with moist sand and provided with two sections cut from a frond of a grasstree on which the female could perch. The sand was watered at least three times per week and the containers were sprayed daily to maintain moisture. Females readily oviposited into the sand cup. Each female received a cotton swab dipped in pollen once a week, mimicking the low pollen availability of the early spring. We estimated the amount of pollen that adhered to cotton swabs by weighing fresh swabs before and after being dipped in pollen, equal to 16.6 ± 0.5 mg (*n* = 20). Females were thereby provided with approximately 2.4 mg pollen per day, which is the intake at which female investment in reproduction falls below that of males and they compete for mating opportunities [53]. In addition, as a source of carbohydrate, once per week females were provided with a cotton swab dipped in glucose solution.

#### 2.3.2. Initial matings

As we were focusing on the fitness benefits gained from multiple mating, all females were allowed to mate without interference on their first mating. Just before the start of each night cycle, a male was introduced to every individual female container. After the lights were turned off, all pairs were monitored continuously for mating activity under red light. When pairs mated, the female was allowed to consume the spermatophylax and the emptied ampulla of the spermatophore as normal. Observations continued until 5 h had passed since the start of the night cycle. Mating activity is restricted to the first 3 h of darkness [45], and the majority of the mating activity in the experiment occurred in the first 2.5 h after the lights had been turned off. The males were removed at the end of the observation period, and if no mating had occurred, the female was introduced to a new male the following night.

#### 2.3.3. Experimental groups

After their first mating, in which all females received both a nuptial gift and sperm from one male, females were randomly assigned to one of three experimental groups: (i) no additional mating: ‘single mating’; (ii) additional mating without spermatophylax: ‘sperm only’; and (iii) additional mating with spermatophylax: ‘gift + sperm’.

Given that this species appears to be facultatively polyandrous [49], and our experiment sought to identify the benefits of multiple mating in polyandrous females, we excluded any female that was unreceptive to multiple mating. Thus, all females were still introduced to males daily, even those in the single mating group, and only the females that reached the *precopula* stage with a second male were used in our analyses. In the single mating group, females were prevented from actually remating by separating the pair immediately after they entered *precopula*. The females in the single mating group were not introduced to males again after reaching their second *precopula*.

Females in the additional mating treatments were allowed to mate up to two more times. *Kawanaphila nartee* females have a minimum refractory period of approximately 4–5 days before they are receptive to remating [52]. Thus, after both the initial and any subsequent matings, we waited for 5 days before introducing the female to a new male. When females in the ‘sperm only’ group remated, we waited until the spermatophore had been transferred, and the male had left the female. Before the female could bend forward to start eating the spermatophylax, we placed her on a small petri dish with thin rolls of modelling clay around the edges. A slightly smaller petri dish was then placed as a lid on top of the female, confining her within a narrow space (approx. 2 mm) so that she was unable to move or reach the spermatophylax. The female was left in this position for 80 min to allow all sperm to transfer from the ampulla. In previous observations, this was found to be the time necessary and sufficient for the female to consume the spermatophylax and for the sperm transfer to finish [52]. After 80 min, the empty spermatophore was removed with forceps, and the female was returned to her container.

In the ‘gift + sperm’ group, females were allowed to consume the spermatophore and its associated spermatophylax freely after each mating. However, to control for the possible effects of confinement, we also placed the females in this group under a petri dish for the same amount of time on the day after the mating took place.

We continued introducing new males daily until 27 October or until the females had mated three times with or without receiving the nuptial gift. After this, females were allowed to continue ovipositing eggs until the end of their lifespan. Since larger females are known to be more fecund [45], pronotum length was measured from all females after they had died, to the nearest 0.1 mm using a binocular microscope fitted with an eyepiece graticule, at 10 times magnification.

The cardboard cups with sand were stored at 20°C and moistened as necessary until eggs were retrieved and counted, approximately 2–3 weeks after the last female died. The number of eggs laid by each female was determined by sieving the sand from the egg cup and dissecting the walls of the cardboard cup under a binocular microscope. The eggs from each female were weighed *en masse* to the nearest 0.1 mg. They were then placed on moistened filter paper in a petri dish that was sealed with parafilm to prevent it from drying and stored at 20°C until egg viability assays were conducted.

#### 2.3.4. Egg viability assay

Attempts to get eggs of *K. nartee* to hatch in laboratory conditions have been unsuccessful because of their prolonged diapause stage, and the unknown environmental cues that are needed to trigger their development in the wild. As an alternative way to measure egg viability, we employed a method based on the assay developed by Philips *et al*. [54] and optimized it for eggs of *K. nartee*. The assay stains the enzyme hexokinase to signal the presence of ATP in viable eggs.

Millipore water (25 µl) was placed in each well of a 96-well polystyrene microplate with flat bottom (Interpath). Then, for each female, 40 eggs were sampled haphazardly from those available, and each egg was placed into an individual well. If the female had laid fewer than 40 eggs, then all the eggs were used. One well was left with just water to act as a blank measurement. As a negative control, three eggs that had been killed by heating at 60°C for 1 h were placed into three more wells. Following egg placement, each egg was macerated in the water using forceps, ensuring thorough cleaning of the forceps between each sample. Once complete, a 2× viability stain was prepared [54] and 25 µl was added to the well and left at room temperature for 1 h. Viability assay components and final assay concentration are reported in the electronic supplementary material, table S1. All components were sourced from Sigma with the exception of the magnesium chloride (Thermofisher). Stain was prepared fresh for each assay. Plates were incubated at room temperature (approx. 20°C) for 1 h before a photograph of the plate was taken, and the image was analysed using ImageJ [55]. A rectangle of stain, free of debris, was selected and the mean pixel values for red, green and blue channels were measured using the RGB Measure plugin for ImageJ. The mean pixel value of the blue channel was divided by the mean pixel value of the green channel to give a quantitative measurement of the colour change after subtracting the blank measurement.

In order to assign an objective threshold absorbance value between ‘viable’ and ‘inviable’ from the measurement of colour change, we pooled values of all the heat-killed control eggs together and determined the upper 95th percentile of their absorbance value. Only eggs that scored above this threshold were considered viable. Given that the eggs of *K. nartee* enter diapause after fertilization, we can only be certain that those scored as viable had been fertilized. The assay cannot distinguish between fertilized eggs that may or may not eventually hatch.

### 2.4. Data handling and analyses

Out of the initial 123 females that mated once and were assigned a treatment, 21 females in the ‘single mating’ group did not enter precopula with a male for a second time, and 29 females in the additional mating groups likewise remained unreceptive to remating. As stated above, these monandrous females were all discarded. Additionally, nine females failed to lay any eggs and were excluded from our analyses. Our final dataset consisted of 18 females in the ‘single mating’ group, 22 females in the ‘sperm only’ group (eight mated twice and 14 mated three times) and 24 females in the ‘sperm + gift’ group (14 mated twice and 10 mated three times). As an exception, when analysing average egg weight, only females that laid five eggs or more were included in the analysis to minimize error in the estimation of egg weight. One female was also excluded from this analysis because her average egg weight showed as a significant outlier, possibly owing to a recording error. In this case, the sample sizes were 15 for the ‘single mating’ group, 19 for the ‘sperm only’ group and 24 for the ‘sperm + gift’ group.

We estimated female lifetime fitness through three key variables: lifetime egg production, average egg weight and egg viability. We also measured female lifespan, to be able to document potential costs of multiple mating [20,56] and account for its effect on the fitness variables measured. Lifetime egg production was the absolute number of eggs collected from the sand and cardboard at the end of the experiment. Average egg weight was measured as the combined weight of all the female’s eggs divided by the number of eggs. For egg viability, we used the proportion of viable eggs in the viability assay. Female lifespan was defined as the number of days from the first mating until death.

In order to determine whether female refractory period was influenced by the additional mating treatments (‘sperm only’ and ‘sperm + gift’), we also investigated how long the females in these groups took to remate. Given that treatments differed only starting from the second mating, we examined the second refractory period, that is, the remating latency between second and third mating. The period for remating latency was defined as the number of days a female was exposed to a new male without mating a third time. For the females that did not mate a third time, a maximum value was assigned as the number of days from the second mating until 27 October, after which females were no longer supplied with males. Excluding females that did not mate a third time from this analysis returned qualitatively similar results.

Statistical analyses were conducted in R (version 4.2.1). The effect of treatment on egg production and egg weight was evaluated with a general linear model (GLM). The full models included treatment, female lifespan, number of matings (one, two or three), the interaction between treatment and number of matings, the interaction between treatment and lifespan and female pronotum length. Each full model was then reduced by ranking all possible models by Akaike information criterion (AIC) values using the ‘dredge’ command (MuMIn package in R [57]) and picking the top model. The effect of treatment on average egg weight was evaluated using a GLM in the same manner as described above. For this, egg weight was transformed into a natural logarithm to meet the assumptions of normality in the residuals. The likelihood of producing viable eggs was evaluated using a generalized linear model with a binomial distribution and a logit-link function with the number of viable and inviable eggs as the dependent variable. Post hoc comparisons between groups were done by examining the overlap of the standard errors (s.e.) of the marginal means from each model using the emmeans package [58]. The effects of treatment on female lifespan and remating latency (for females that mated more than once) were evaluated with a GLM and a generalized linear model (negative binomial distribution), respectively, with only pronotum length as a covariate.

## 3. Results

**Figure 2.**
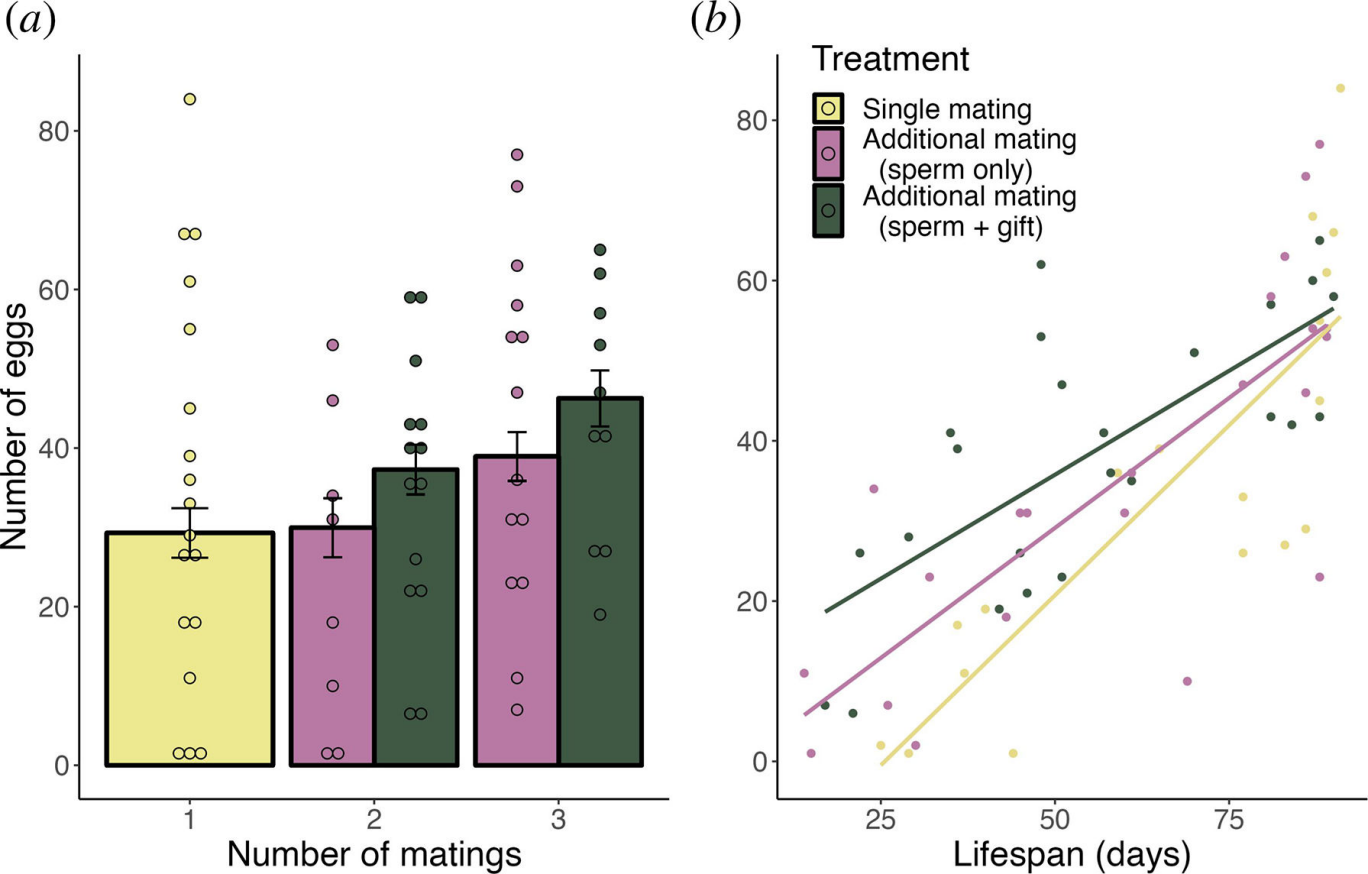
(*a*) Estimated marginal means of the female’s relative lifetime egg production by treatment and number of matings. Error bars represent ± s.e. (*b*) Female egg production as an interaction between treatment and lifespan (number of days alive since first mating).

The best model predicting lifetime egg production (GLM, d.f. = 57) included treatment, female lifespan, number of matings and the interaction between treatment and lifespan (table 1). Egg production was elevated by additional matings, both in terms of additional ejaculates and additional nuptial gifts, with the highest egg output shown by females that mated three times and were allowed to consume the spermatophylax (figure 2*a*). However, the interaction between treatment and lifespan showed that the treatment effects were most pronounced in females with shorter lifespans (figure 2*b*).

**Table 1.** Results of (*a*) general linear models on number of eggs, egg weight and lifespan and (*b*) generalized linear models on egg viability (binomial model) and remating latency (negative binomial model).

(*a*)	d.f.	F-value	*p*
number of eggs			
treatment	1	4.03	0.049
lifespan	1	94.70	<0.001
*n* of matings	1	5.17	0.027
treatment × lifespan	2	1.91	0.157
egg weight			
lifespan	1	40.18	<0.001
lifespan			
treatment	2	0.88	0.4207
pronotum length	1	2.34	0.131

(*b*)		*χ*²	*p*
egg viability			
treatment	1	0.07	0.791
lifespan	1	33.12	<0.001
*n* of matings	1	5.52	0.019
pronotum length	1	3.83	0.05
treatment × *n* of matings	1	5.23	0.022
remating latency			
treatment	1	5.07	0.024
prototum length	1	1.45	0.228

Additional mating with or without spermatophylax consumption had no significant effects on average egg weight. The best model predicting average egg weight only included female lifespan (GLM, d.f. = 56; table 1). Long-lived females produced heavier eggs in general (electronic supplementary material, figure S1).

**Figure 3.**
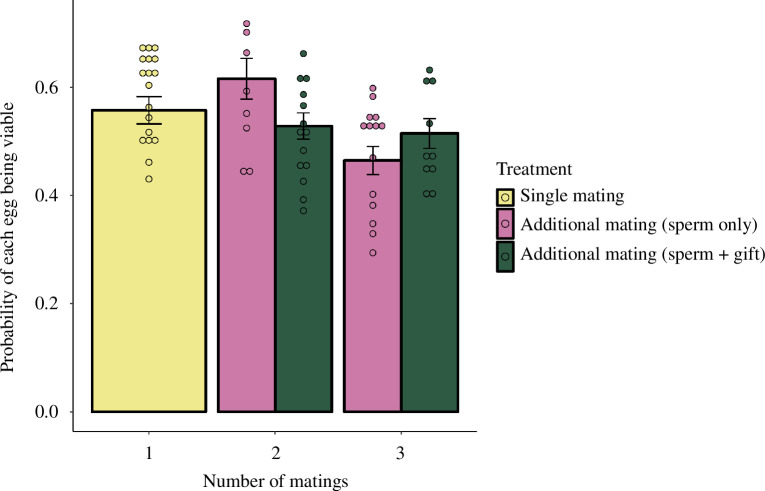
Estimated marginal means of predicted probability of viable eggs by treatment and number of matings. Error bars represent ± s.e. and data points are predicted values from the final model.

The probability of each egg being viable was influenced significantly by the number of matings and its interaction with treatment. The best model (binomial GLM, d.f. = 57) included treatment, female lifespan, number of matings, female pronotum length and the interaction between treatment and number of matings (table 1; figure 3). Post hoc comparisons suggest that three times-mated females had reduced egg viability compared with twice-mated females only when they were prevented from eating the spermatophylax.

**Figure 4.**
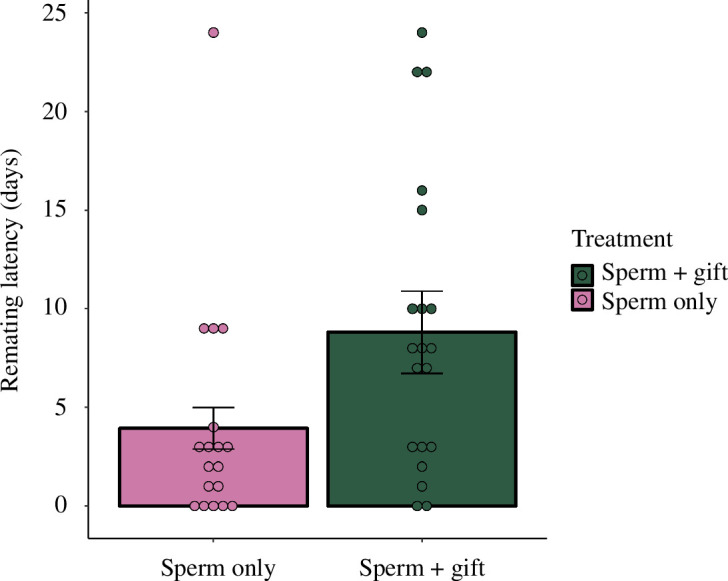
Estimated marginal means of the number of days the females were exposed to a new male without mating a third time, depending on the treatment that was applied on their second mating. Error bars represent ± s.e.

Treatment did not have a significant effect on female lifespan (GLM, d.f. = 60; table 1). Spermatophylax consumption had a significant effect on female remating latency. Females that were prevented from eating the spermatophylax remained unreceptive for an average of 3.9 days after they were introduced to a male, while females that ingested a spermatophylax remained unreceptive for an average 8.8 days (negative binomial GLM, d.f. = 35; table 1; figure 4).

## 4. Discussion

The circumstances that lead to intrasexual mating competition among females, and whether female–female competition should be considered sexual selection, remain under debate [3–6,8,10,59]. While females compete over access to mates in many species, Shuker & Kvarnemo [10] suggested that this constitutes natural or sexual selection depending largely on whether the benefits gained from additional matings come from the gametes or other resources obtained. In this study, we attempted to disentangle the benefits obtained by females from multiple mating by female *K. nartee*, by manipulating the number of ejaculates received from different males and the number of nutritional gifts. We show that additional ejaculates can increase egg production, and this effect was further elevated by the consumption of additional nuptial gifts. The effects of multiple mating were most pronounced in short-lived females. However, multiple mating did not have a clear positive effect on the weight or viability of the eggs. Below, we argue that elevated egg laying in early life can have the strongest fitness effects on female *K. nartee* and thus can lead to female mating competition for access to multiple matings.

According to Shuker & Kvarnemo’s [10] suggested definition of sexual selection, only competition for a limited availability of opposite-sex gametes for fertilization should be considered sexual selection. Nuptial gifts complicate this distinction, because the acquisition of gametes and resources are inextricably linked, and females might compete for both resources and gametes simultaneously [60]. Our study asked whether female remating provided fitness benefits from the additional nutrients derived from the spermatophylaces they received from multiple males or the additional gametic material from multiple ejaculates. If we assume that female fitness is determined largely by lifetime egg production, our results suggest that both additional ejaculates from more males and additional nutrition from their nuptial gifts provide the female fitness benefits, and the effects of ejaculates and nutrients are additive. Therefore, under Shuker & Kvarnemo’s [10] definition, female mating competition in *K. nartee* should result in both sexual and natural selections.

Our results are in line with a previous study of this species, which showed that nuptial gift consumption increased female egg production soon after mating [43]. More generally, they are also in line with many other studies that have found that egg production is increased by additional matings in a number of species (reviewed in [20,33]) and by additional nuptial gifts [34,35]. The studies of Orthoptera that have found fitness benefits associated with spermatophylax feeding have usually limited female access to a resource that is contained in the spermatophylax, such as protein or water [35,36]. By contrast, when abundant resources have been provided, studies of these same species have found no fitness increase from nuptial gift consumption [61,62]. These studies suggest that selection on female mating frequency is likely to be context-dependent, and that females are selected for a higher mating frequency when they are resource-limited [38]. Our study mimicked the limited diet that characterizes early spring for our experimental females, which is when pollen resources are scarce and female–female competition occurs naturally [42].

We found that the differences between treatments in egg production were most pronounced among females with shorter lifespans. There are several potential explanations for this pattern. First, females that died early might have been in poor condition, and the relative benefits of additional nutrients and ejaculates might have been greater for these females. An interaction between body condition and spermatophylax consumption has rarely been studied directly, but studies showing that resource-limited females are more polyandrous and gain more benefits from nuptial gifts allude to this potential effect [35,36,61]. Moreover, average egg weight was positively correlated with female lifespan in our study. Since both traits are likely to be influenced by female condition, the underlying reason for their positive correlation could be their mutual dependency on condition. Second, polyandry has often been shown to lower female lifespan, while egg production is simultaneously increased [20,33,63]. Female lifespan was unaffected by multiple mating in our study, but engaging in breeding has previously been shown to result in higher mortality in *K. nartee*, especially for females breeding under food limitation [56]. Therefore, it is also possible that compounds in the ejaculate could increase female egg production at the cost of female longevity, similar to some other insect species [64–66]. In our experiment, females in all treatments appeared to achieve equivalent lifetime fecundity if they were long-lived; accumulating a greater amount of pollen over a longer life might allow single mating females to achieve a similar fecundity to multiple mating females (figure 1*b*). However, the lifespans of females in our study are unlikely to reflect natural conditions. Females in our experiment were held in optimal conditions in terms of temperature and water availability, and they were not exposed to potential predators. *Kawanaphila nartee* adults only live for one breeding season, and the population typically experiences mass mortality following just 1–2 hot and dry days in the late spring. In 2022, when this experiment was conducted, these conditions were already met in early November, whereas a large proportion of our laboratory-kept females lived until the first half of December. Thus, the lifespans recorded in our study were approximately a third longer than they would have been in the wild, and individuals that lived approximately 60 days or less (figures 1 and 2*b*) may be more representative of natural populations. Therefore, investing in a longer lifespan is probably not beneficial for *K. nartee*, and offspring production in early life may better reflect patterns of lifetime fitness in nature.

Multiple mating and spermatophylax consumption did not clearly increase the viability of eggs. Rather, there was a significant interaction, whereby egg viability was broadly similar across treatments, but females that only received additional ejaculates had a decrease in egg viability if they mated three times (figure 2). This pattern could be explained if compounds in the ejaculate stimulate females to increase their egg production, as discussed above [67]. When females are deprived of eating the spermatophylax, they may lack the resources necessary to sustain increased egg production after several matings and thus produce a lower proportion of viable eggs [68,69]. While our egg viability measurements should be adequate for estimating the proportion of fertilized eggs that enter diapause, differences in embryo mortality may also occur further down the developmental pathway [26], so that our study may underestimate any effects multiple mating might have on hatching success.

In many insects, especially *Drosophila*, seminal fluid in the male ejaculate contains proteins or other compounds that are absorbed through the female genital tract, some of which affect egg production [66,67,70,71]. Shuker & Kvarnemo’s [10] definition does not address this phenomenon, and our study is unable to disentangle the fitness effects of seminal fluid and sperm components of the ejaculate. This raises the question of whether the ejaculate should be considered as a resource or as part of the gametes. Male gametes never enter the female as single cells but are always encompassed by other elements of the seminal fluid [72], many of which may be essential for fertilization. Seminal compounds can often enhance the viability of sperm, for example, by facilitating the storage of sperm in the female reproductive tract or by directly enhancing fertilization competency of sperm and zygote viability [73–75]. Since successful fertilization is not usually possible without seminal fluids, one can argue that seminal fluid compounds entering the female genital tract within the ejaculate should be included as part of the ‘gametes’ in the definition for sexual selection, if they are directly involved in fertilization and offspring development.

It has been suggested that nuptial gifts may have evolved to allow males to manipulate the duration of sperm transfer, increase the female refractory period or to maximize egg production before the female remates [30,33,64], and that females would only later have co-evolved to metabolize some of these compounds for their benefit. Our finding that spermatophylax consumption did not increase egg weight or viability and that it led to a longer refractory period, could be seen to support this hypothesis. However, there are alternative explanations for these patterns. A previous study of *K. nartee* showed that females which were prevented from consuming the spermatophylax on their initial mating laid smaller eggs [43]. This contrasts with our results and may indicate that more of the nutrients in the spermatophylax get allocated to egg weight on the first mating, than in subsequent matings. We also showed that females which were allowed to consume the spermatophylax waited longer to remate, which confirms the pattern from a previous study [52]. However, in Simmons & Gwynne [52], females prevented from eating the spermatophylax only had a shorter refractory period under a limited diet (as imposed in our study), suggesting that female receptivity to remating is motivated by female hunger and not purely by male manipulation. Taken together, females seem able to circumvent male attempts to manipulate their receptivity via compounds transferred in the ejaculate and instead remate at intervals that optimizes their own fitness [76]. It may be that instead of allocating the nutrients of the spermatophylax to longevity or the production of more viable eggs, the females use the energy from the nuptial gift to boost their metabolism to produce more eggs [77].

Given our findings for *K. nartee*, how does the definition of sexual selection proposed by Shuker & Kvarnemo [10] affect our view of the mating systems in this and other nuptial feeding species? Nuptial gifts are a common feature of arthropod taxa, and their potential fitness benefits have been evaluated in many of these groups [78], including crickets [35,36,39,40,61,79], scorpionflies [34], beetles [37] and spiders [38,80,81]. However, despite evidence that polyandry or ingestion of multiple nuptial gifts can increase female fitness in many of these species [34–36,40,80], descriptions of intrasexual female mating competition or male mate choice still remain uncommon among them [82]. At the same time, exaggerated female traits that are used in mating competition have only been described in nuptial-feeding insect taxa, such as dance flies from the genera *Empis* and *Rhamphomyia* [83–85], and Orthopterans such as *K. nartee* [48,49]. Given that mating competition is expensive, females competing over the opportunity to mate multiple times, especially when the availability of males is already low, would be expected to evolve only if the benefits gained from each mating exceed mating costs. However, our results show that the benefits *K. nartee* females gain from multiple mating are similar to other species that do not show evidence of intrasexual female mating competition, so there may be additional factors that intensify mate limitation in this species. It seems that intense female mating competition occurs mainly when the nutrient limitation of the environment, combined with high costs of nuptial gift production [43], limit the availability of males. This will bias the operational sex ratio towards an excess of females [42,86] and increase the chance of mating failure among females [19]. It is thus likely it is the combination of mate limitation resulting from these conditions, as well as the benefits gained from additional ejaculates and spermatophylaces, that drives the evolution of female competition. In this case, female *K. nartee* and other species with a similar mating system would still be considered to be limited by gametes, and thus, under sexual selection.

In conclusion, our results show that female *K. nartee* lifetime egg production can benefit from polyandry, both in terms of additional ejaculates and nutrition gained from nuptial gifts, and that these effects seem to be additive. However, we did not find evidence that multiple mating would increase egg weight or viability, or affect lifespan. Given the short life of adult *K. nartee* in natural conditions, we argue that female fitness is probably predicted by elevated relative lifetime egg production. We thus conclude that following Shuker & Kvarnemo’s [10] definition, female mating competition over additional matings falls under an inextricably linked combination of sexual and natural selection. This probably applies to other nuptial-feeding species as well, where resource limitation reduces the availability of mates.

## Data Availability

Data and relevant R code for this research are available in the Dryad data repository [87]. Electronic supplementary material is available online [88].
